# Exogenous hormone use, reproductive factors and risk of intrahepatic cholangiocarcinoma among women: results from cohort studies in the Liver Cancer Pooling Project and the UK Biobank

**DOI:** 10.1038/s41416-020-0835-5

**Published:** 2020-05-07

**Authors:** Jessica L. Petrick, Úna C. McMenamin, Xuehong Zhang, Anne Zeleniuch-Jacquotte, Jean Wactawski-Wende, Tracey G. Simon, Rashmi Sinha, Howard D. Sesso, Catherine Schairer, Lynn Rosenberg, Thomas E. Rohan, Kim Robien, Mark P. Purdue, Jenny N. Poynter, Julie R. Palmer, Yunxia Lu, Martha S. Linet, Linda M. Liao, I-Min Lee, Jill Koshiol, Cari M. Kitahara, Victoria A. Kirsh, Jonathan N. Hofmann, Barry I. Graubard, Edward Giovannucci, J. Michael Gaziano, Susan M. Gapstur, Neal D. Freedman, Andrea A. Florio, Dawn Q. Chong, Yu Chen, Andrew T. Chan, Julie E. Buring, Laura E. Beane Freeman, Jennifer W. Bea, Christopher R. Cardwell, Peter T. Campbell, Katherine A. McGlynn

**Affiliations:** 1 Division of Cancer Epidemiology and Genetics, National Cancer Institute, Bethesda, MD USA; 2 Slone Epidemiology Center, Boston University, Boston, MA USA; 3 Centre for Public Health, Queen’s University Belfast, Belfast, Northern Ireland UK; 4 Channing Division of Network Medicine, Brigham and Women’s Hospital, Boston, MA USA; 5 Department of Population Health, New York University School of Medicine, New York, NY USA; 6 NYU Perlmutter Cancer Center, New York University School of Medicine, New York, NY USA; 7 Department of Epidemiology and Environmental Health, University at Buffalo, Buffalo, NY USA; 8 Division of Gastroenterology, Massachusetts General Hospital and Harvard Medical School, Boston, MA USA; 9 Department of Epidemiology, Harvard T.H. Chan School of Public Health, Boston, MA USA; 10 Division of Preventive Medicine, Department of Medicine, Brigham and Women’s Hospital, Boston, MA USA; 11 Department of Epidemiology & Population Health, Albert Einstein College of Medicine, Bronx, NY USA; 12 Department of Exercise and Nutrition Sciences, Milken Institute School of Public Health, George Washington University, Washington, DC USA; 13 Division of Pediatric Epidemiology and Clinical Research and Masonic Cancer Center, University of Minnesota, Minneapolis, MN USA; 14 Department of Population Health and Disease Prevention, Program in Public Health, University of California, Irvine, Irvine, CA USA; 15 Epidemiology Division, Dalla Lana School of Public Health, University of Toronto, Toronto, Ontario, Canada; 16 VA Boston Healthcare System, Boston, MA USA; 17 Behavioral and Epidemiology Research Group, American Cancer Society, Atlanta, GA USA; 18 Division of Medical Oncology, National Cancer Centre, Singapore, Singapore; 19 Department of Environmental Medicine, New York University School of Medicine, New York, NY USA; 20 Clinical and Translational Epidemiology Unit, Massachusetts General Hospital, Boston, MA USA; 21 Department of Medicine, University of Arizona, Tucson, AZ USA

**Keywords:** Risk factors, Bile duct cancer

## Abstract

**Background:**

Intrahepatic cholangiocarcinoma (ICC) arises from cholangiocytes in the intrahepatic bile duct and is the second most common type of liver cancer. Cholangiocytes express both oestrogen receptor-α and -β, and oestrogens positively modulate cholangiocyte proliferation. Studies in women and men have reported higher circulating oestradiol is associated with increased ICC risk, further supporting a hormonal aetiology. However, no observational studies have examined the associations between exogenous hormone use and reproductive factors, as proxies of endogenous hormone levels, and risk of ICC.

**Methods:**

We harmonised data from 1,107,498 women who enroled in 12 North American-based cohort studies (in the Liver Cancer Pooling Project, LCPP) and the UK Biobank between 1980–1998 and 2006–2010, respectively. Cox proportional hazards regression models were used to generate hazard ratios (HR) and 95% confidence internals (CI). Then, meta-analytic techniques were used to combine the estimates from the LCPP (*n* = 180 cases) and the UK Biobank (*n* = 57 cases).

**Results:**

Hysterectomy was associated with a doubling of ICC risk (HR = 1.98, 95% CI: 1.27–3.09), compared to women aged 50–54 at natural menopause. Long-term oral contraceptive use (9+ years) was associated with a 62% increased ICC risk (HR = 1.62, 95% CI: 1.03–2.55). There was no association between ICC risk and other exogenous hormone use or reproductive factors.

**Conclusions:**

This study suggests that hysterectomy and long-term oral contraceptive use may be associated with an increased ICC risk.

## Background

Intrahepatic cholangiocarcinoma (ICC) rates have been rapidly increasing in the US since the mid-1980s.^1^ Between 2001 and 2016, ICC rates among US women more than doubled (from 0.6 to 1.4/100,000 person-years, respectively).^2^ ICC arises from cholangiocytes in the intrahepatic bile duct and is the second most common type of liver cancer in the US, accounting for 12% of primary liver cancers.^3^

As cholangiocytes express both oestrogen receptor-α and -β,^4–6^ hormonal factors have been hypothesised to play a role in cholangiocarcinogenesis.^7^ Experimental evidence suggests that oestrogen promotes cholangiocarcinogenesis, while selective oestrogen receptor modulators can inhibit growth.^8–10^ Additionally, a prior study from Khon Kaen, Thailand, reported higher circulating oestradiol in men with cholangiocarcinoma compared to controls.^11^ Another recent study from the Liver Cancer Pooling Project (LCPP) reported that higher levels of circulating oestradiol in women were associated with an increased ICC risk.^12^ Taken together, these results suggest that factors that affect oestrogenic regulation may play a role in the aetiology of ICC.

Several epidemiologic studies have examined exogenous hormone use and reproductive factors in relation to all primary liver cancer or hepatocellular carcinoma (HCC) risk,^7,13^ but not ICC risk alone. One recent study from Sweden found that menopausal hormone therapy (MHT) use was associated with a lower risk of gastrointestinal cancers, but there were too few cases to examine the association with ICC as a unique outcome.^14^ Thus, we conducted a prospective analysis of North American and European women to examine exogenous hormonal exposures and reproductive factors, as proxies of endogenous hormonal exposures, in relation to ICC risk.

## Methods

### Study population

For this study, we combined the resources of the National Cancer Institute (NCI) Cohort Consortium and the UK Biobank. All North American-based cohort studies that are members of the NCI Cohort Consortium were invited to participate in the LCPP, as previously described.^13^ For the current study, which is restricted to women, 12 cohorts contributed information on exogenous hormones and/or reproductive factors and ICC cases: NIH-AARP Diet and Health Study (AARP),^15^ Agricultural Health Study (AHS),^16^ The Breast Cancer Detection Demonstration Project (BCDDP),^17^ Prostate, Lung, Colorectal and Ovarian Cancer Screening Trial (PLCO),^18^ Women’s Health Study (WHS),^19^ New York University Women’s Health Study (NYUWHS),^20^ Cancer Prevention Study–II (CPS-II) Nutrition Cohort,^21^ Iowa Women’s Health Study (IWHS),^22^ Black Women’s Health Study (BWHS),^23^ Women’s Health Initiative (WHI),^24^ Nurses’ Health Study (NHS),^25^ and the Canadian Study of Diet, Lifestyle, and Health (CSLDH)^26^ (Supporting Table S1). The Physicians’ Health Study (PHS)^27^ and Health Professionals Follow-Up Study (HPFS)^28^ were excluded, as these cohorts only included men. The US Radiologic Technologists (USRT) Study^29^ is also excluded, as this cohort had no women ICC cases. The UK Biobank is a large prospective cohort study that recruited over 500,000 men and women aged 40–69 years from one of 22 assessment centres located across England, Scotland and Wales between 2006 and 2010.^30^ All studies participating in the LCPP contributed de-identified data following data sharing agreements between NCI and the cohort’s academic institute; the UK Biobank was approved by the North West Multi-Centre Research Ethics Committee, and all participants provided written informed consent.

With the exception of the CSLDH, all other studies contributed the full cohort of individuals. For cost-efficiency, CSLDH was performed using a case-cohort design. Thus, from the entire cohort of women in CSLDH (*n* = 39,618) at baseline, an age-stratified random sample was selected to create a sub-cohort of women (*n* = 3224), which are utilised in analyses to represent the full cohort.^31^ In total, 1,107,498 women participants were included: 851,157 women from the LCPP and 256,341 women from the UK Biobank.

### Outcomes

Incident primary liver cancer (defined as International Classification of Diseases, 10th edition [ICD-10] diagnostic code C22) was ascertained by linkage to state-, provincial- or country-specific cancer registries or medical/pathology record review; dates of follow-up are given for each study in Supplemental Table S1. Cases of ICC were classified using the ICD-O-3 morphology codes 8032–8033, 8041, 8050, 8070–8071, 8140–8141, 8160, 8260, 8480, 8481, 8490 and 8560. The current study included 237 ICC cases (LCPP *n* = 180 and UK Biobank *n* = 57) and 1,107,261 non-cases.

### Exposures

In the LCPP, reproductive factors were self-reported via hard copy questionnaire and included age at menarche, parity, age at first birth, age at natural menopause, oophorectomy, fertile duration (years between menarche and menopause), oral contraceptive use, duration of oral contraceptive use, MHT use, recency of MHT use (never, current, former), duration of MHT use, type of MHT use (none, oestrogen-only MHT, oestrogen-progesterone combination MHT, other), and MHT route of administration (none, oral, non-oral). In the UK Biobank, the same exposures were assessed in a similar self-reported manner via touchscreen computerised and interviewer-administered questionnaires at baseline. The exception to similar exposure ascertainment is type of MHT, which was only asked of women who reported ‘current’ MHT use at baseline and was verified during a verbal interview with a UK Biobank nurse.

### Statistical analysis

Cox proportional hazards regression models were used to estimate hazard ratios (HRs) and 95% confidence intervals (CIs) for the associations between exogenous hormone use and reproductive factors and risk of ICC. To account for the case-cohort design of CSLDH in the LCPP, all cases were weighted as one, and non-cases were weighted according to the inverse of their stratum-specific sampling fractions. All participants in the remaining cohorts were weighted as one using PROC SURVEYPHREG.^32^ Follow-up of the analytic cohort occurred from time of exposure ascertainment until an event (i.e., incident ICC) or right-censoring (i.e., death, loss to follow-up, or last date of follow-up), whichever occurred first. The proportional hazards assumption was tested using an interaction between exogenous hormones or reproductive factors with log(time) in models that included confounders; no violations were observed (*p* > 0.05). The adjusted study-specific effect estimates from the LCPP and the UK Biobank were combined using fixed-effects meta-analytic models.

Based on existing literature, LCPP models contained the following potential confounders:^33^ age (continuous), alcohol consumption (g/day: none, ≤1.08, >1.08–3.58, >3.58–13.54, >13.54), body mass index (BMI, <25, 25–29.9, ≥30 kg/m^2^), diabetes (yes, no), race (white, other), smoking (never, former, current), parent cohort study, and education (<high school, high school, some college/vocational, college, graduate degree). UK Biobank models contained age (continuous), alcohol consumption [never, former, current light/occasional (<16 g/day), current heavy (≥16 g/day)], BMI (<25, 25–29.9, ≥30 kg/m^2^), smoking (never, former, current), and education (<secondary school, secondary school, college, graduate degree). Models using UK Biobank data were not adjusted for race, as almost 95% of the cohort is white, nor diabetes, as only one ICC case had a type 2 diabetes diagnosis. In the UK Biobank, medical history of hepatitis B and C virus (HBV/HCV) and cirrhosis were examined as potential covariates, but they did not alter the estimates and are not included in the final models (data not shown). Models examining reproductive factors also contained menopausal status (pre-, post-menopausal), while models examining menopausal factors (i.e., age at menopause and MHT use) were restricted to post-menopausal women. A lag analysis, excluding the first two years of follow-up, was performed to account for potential pre-existing liver disease. Analyses were conducted using SAS version 9.4 (SAS Institute, Cary, NC) and STATA version 14 (StataCorp LP, College Station, TX).

### Nested case-control study of HBV/HCV

In the LCPP, 47 ICC cases that had a serum sample available were tested for determination of HBV and HCV serology status, in addition to 98 matched controls. To determine HBV status, hepatitis B surface antigen (HBsAg) was assayed using the Bio-Rad GS HBsAg 3.0 enzyme immunoassay (Bio-Rad Laboratories, Redmond, WA, USA). To determine HCV status, antibody to hepatitis C virus (anti-HCV) was assessed using the Ortho HCV Version 3.0 ELISA test system (Ortho-Clinical Diagnostics, Inc., Raritan, NJ, USA).

## Results

Participants averaged 13.0 years of follow-up (maximum 30.4 years) in the LCPP and 5.5 years of follow-up (maximum 8.5 years) in the UK Biobank. Table 1 summarises women participant characteristics, which were similar between the LCPP and the UK Biobank. For example, the mean ages of non-cases were 57.7 and 56.1 and cases were 61.9 and 60.3 years, respectively. Among the non-cases, there was a similar prevalence of non-smokers (51.0 vs. 59.6%) and individuals with a BMI ≥ 30 kg/m^2^ (20.4 vs. 23.9%) in the LCPP and the UK Biobank, respectively. However, the LCPP had more post-menopausal non-cases (83.0 vs. 59.6%). In both the LCPP and the UK Biobank, ICC cases were more likely to be post-menopausal, to be current or past smokers, and to have a BMI ≥ 30 kg/m^2^.

**Table 1:** Characteristics of female participants in the Liver Cancer Pooling Project and the UK Biobank.

	Liver Cancer Pooling Project		UK Biobank	
	No. non-cases (*N* = 850,977)	No. ICC cases (*N* = 180)	No. non-cases (*N* = 256,284)	No. ICC cases (*N* = 57)
	No. (%)	No. (%)	No. (%)	No. (%)
Age at baseline, mean (SD)	57.7 (10.56)	61.9 (7.20)	56.1 (0.02)	60.3 (0.87)
<50	153,709 (18.1)	9 (5.0)	62,807 (24.5)	5 (8.8)
50–59	286,553 (33.7)	52 (28.9)	88,718 (34.6)	19 (33.3)
60–69	332,574 (39.1)	97 (53.9)	103,704 (40.5)	31 (54.4)
≥70	78,141 (9.2)	22 (12.2)	1,055 (0.4)	2 (3.5)
Menopausal status				
Pre-menopausal	134,595 (15.8)	9 (5.0)	62,992 (24.6)	4 (7.0)
Post-menopausal	706,218 (83.0)	171 (95.0)	152,752 (59.6)	41 (71.9)
Missing	10,164 (1.2)	0 (0.0)	40,540 (15.8)	12 (21.1)
Alcohol intake				
Liver Cancer Pooling Project				
Non-drinker	258,141 (30.3)	48 (26.7)		
Quartile 1: ≤1.08 g/day	170,803 (20.1)	41 (22.8)		
Quartile 2: 1.09–3.58 g/day	152,765 (18.0)	32 (17.8)		
Quartile 3: 3.59–13.54 g/day	129,502 (15.2)	29 (16.1)		
Quartile 4: >13.54 g/day	98,621 (11.6)	21 (11.7)		
Missing	41,145 (4.8)	9 (5.0)		
UK Biobank				
Never			15,072 (5.9)	4 (7.0)
Current (<16 g/day)			167,319 (65.3)	39 (68.4)
Current (≥16 g/day)			62,762 (24.5)	10 (17.5)
Former			9251 (3.6)	4 (7.0)
Missing			1,880 (0.7)	0 (0.0)
Smoking status				
Never	434,198 (51.0)	80 (44.4)	152,637 (59.6)	20 (35.1)
Former	284,799 (33.5)	75 (41.7)	79,316 (30.9)	29 (50.9)
Current	114,922 (13.5)	23 (12.8)	22,922 (8.9)	8 (14.0)
Missing	17,058 (2.0)	2 (1.1)	1409 (0.6)	0 (0.0)
BMI status (kg/m^2^)				
<25	387,573 (45.5)	59 (32.8)	101,363 (39.6)	22 (38.6)
25–29.9	260,561 (30.6)	67 (37.2)	92,241 (36.0)	20 (35.1)
≥30	173,376 (20.4)	45 (25.0)	61,324 (23.9)	15 (26.3)
Missing	29,467 (3.5)	9 (5.0)	1,356 (0.5)	0 (0.0)
Diabetes				
No	792,844 (93.2)	159 (88.3)	247,484 (96.6)	56 (98.3)
Yes	45,678 (5.4)	18 (10.0)	8800 (3.4)	1 (1.7)
Missing	12,455 (1.5)	3 (1.7)	0 (0.0)	0 (0.0)
Race				
White	715,739 (84.1)	156 (86.7)		
Black	93,734 (11.0)	9 (5.0)		
Asian/Pacific Islander	11,297 (1.3)	4 (2.2)		
American Indian/Alaskan Native	2,032 (0.2)	0 0.0		
Other	19,336 (2.3)	7 (3.9)		
Missing	8,839 (1.0)	4 (2.2)		
Education				
High School or Less	45,659 (5.4)	12 (6.7)		
High School	184,586 (21.7)	32 (17.8)	73,038 (28.5)	10 (17.5)
Some College/Vocational	257,563 (30.3)	59 (32.8)		
College Degree	190,027 (22.3)	44 (24.4)	41,596 (16.2)	8 (14.0)
Graduate Degree	135,288 (15.9)	25 (13.9)	94,233 (36.8)	18 (31.6)
None of the above			42,439 (16.6)	20 (35.1)
Missing	37,854 (4.4)	8 (4.4)	4978 (1.9)	1 (1.8)

As shown in Table 2, there were null associations between age at menarche, parity, or age at first birth and risk of ICC. Similarly, there was a null association with ever use of oral contraceptives (HR = 1.12, 95% CI: 0.82–1.53). However, examining duration of oral contraceptive use, nine or more years of use was associated with 62% increased risk of ICC in the combined study population (HR = 1.62, 95% CI: 1.03–2.55), which was in the same direction in both LCPP (HR = 1.71, 95% CI: 1.01–2.89) and UK Biobank cohorts (HR = 1.38, 95% CI: 0.56–3.39).

**Table 2:** Association between reproductive factors and intrahepatic cholangiocarcinoma in the Liver Cancer Pooling Project and the UK Biobank.

	Liver Cancer Pooling Project					UK Biobank					Combined		
Reproductive Factors	No. non-cases (*N* = 722,150)^a^	No. ICC Cases (*N* = 154)^a^	HR^b^	95% CI		No. non-cases (*N* = 209,464)^a^	No. ICC Cases (*N* = 44)^a^	HR^c^	95% CI		HR	95% CI	
Age at menarche													
<12	207,893	49		Referent		39,215	4		Referent			Referent	
12–13	366,443	72	0.73	0.50–1.07		89,034	23	2.59	0.89–7.51		0.84	0.59–1.20	
14+	133,893	29	0.76	0.48–1.20		75,233	16	1.83	0.61–5.52		0.87	0.57–1.32	
Missing	13,921	4				5,982	1						
* p* for trend				0.72					0.36				
Ever had children													
No	98,304	20		Referent		40,205	6		Referent			Referent	
Yes	611,875	134	0.87	0.54–1.39		169,121	38	1.14	0.47–2.73		0.92	0.61–1.41	
Missing	11,971	0				138	0						
Number of children													
0	97,651	20		Referent		40,205	6		Referent			Referent	
1	78,487	12	0.67	0.33–1.37		28,182	7	1.37	0.46–4.09		0.83	0.46–1.50	
2	185,348	38	0.83	0.48–1.44		91,323	18	1.05	0.41–2.67		0.88	0.55–1.41	
3+	345,531	83	0.82	0.50–1.35		49,752	13	1.16	0.43–3.13		0.88	0.56–1.37	
Missing	15,133	1				0	0						
* p* for trend				0.97					0.35				
Age at first birth (parous women)													
<21	105,544	19		Referent		19,734	5		Referent			Referent	
21–24	275,185	72	1.31	0.77–2.21		40,407	8	0.88	0.28–2.73		1.22	0.75–1.98	
25–28	147,059	25	0.74	0.39–1.39		44,890	9	1.26	0.40–3.95		0.84	0.48–1.47	
≥29	59,050	15	1.02	0.49–2.11		35,669	9	1.87	0.57–6.16		1.21	0.65–2.25	
Missing	135,312	23				28,419	7						
* p* for trend				0.62					0.90				
Oral contraceptive use													
No	384,699	97		Referent		39,095	9		Referent			Referent	
Yes	331,664	57	1.06	0.75–1.48		169,887	35	1.46	0.68–3.16		1.12	0.82–1.53	
Missing	5,787	0				480	0						
Duration of oral contraceptive use													
None	384,699	97		Referent		39,095	9		Referent			Referent	
<1 year	66,782	12	0.94	0.52–1.69		7,039	2	1.55	0.33–7.24		1.00	0.58–1.74	
1–2.5 years	77,857	12	1.09	0.59–2.01		17,999	3	0.92	0.25–3.45		1.06	0.61–1.84	
2.5–6 years	50,176	5	0.59	0.24–1.49		22,069	7	2.00	0.72–5.51		1.01	0.51–1.98	
6–9 years	70,620	11	1.00	0.54–1.88		22,277	3	0.94	0.25–3.59		0.99	0.57–1.73	
9+ years	61,781	17	1.71	1.01–2.89		78,287	14	1.38	0.56–3.39		1.62	1.03–2.55	
Missing	10,235	0				22,696	6						
* p* for trend				0.14					0.46				

^a^Numbers are for women with non-missing covariates.

^b^Adjusted for age (continuous), alcohol (g/day: none, ≤1.08, >1.08–3.58, >3.58–13.54, >13.54), BMI (kg/m^2^: <25, 25–29.9, ≥30), diabetes (yes, no), race (white, other), smoking (never, former, current), parent cohort study, menopausal status (pre-, post-menopausal), and education (<high school, high school, some college/vocational, college, graduate degree).

^c^Adjusted for age (continuous), alcohol (never, former, current light/occasional (<16 g/day), current heavy (≥16 g/day)), BMI (kg/m^2^: <25, 25–29.9, ≥30), smoking (never, former, current), menopausal status (pre-, post-menopausal), and education (<secondary school, secondary school, college, graduate degree).

There was no association with age at natural menopause, but there was a 2-fold increased risk of ICC associated with hysterectomy (HR = 1.98, 95% CI: 1.27–3.09), compared to women aged 50–54 at natural menopause, which was driven by the results from the LCPP (HR = 2.25, 95% CI: 1.38–3.65; Table 3). Further adjustment in the LCPP for MHT use did not substantially affect the estimate (HR = 2.15, 95% CI: 1.31–3.52), although women with hysterectomy were more likely to report MHT use—especially oestrogen-only MHT (Supplemental Table S2). There was no association with total fertile duration. Examining MHT use, including recency, duration, or route of administration, revealed no associations with ICC risk. However, there was a possible indication of increased risk of ICC and oestrogen-only therapy in post-menopausal women (HR = 1.44, 95% CI: 0.91–2.28).

**Table 3:** Associations between menopausal factors and menopausal hormone therapy (MHT) use among post-menopausal women and risk of intrahepatic cholangiocarcinoma in the Liver Cancer Pooling Project and the UK Biobank.

	Liver Cancer Pooling Project					UK Biobank					Combined		
Age at menopause	No. non-Cases (*N* = 722,150)^a^	No. ICC Cases (*N* = 154)^a^	HR^b^	95% CI		No. non-Cases (*N* = 209,464)^a^	No. ICC Cases (*N* = 44)^a^	HR^c^	95% CI		HR	95% CI	
Natural menopause													
<45	35,745	7	0.82	0.37–1.81		11,273	14	1.21	0.38–3.84		0.93	0.48–1.79	
45–49	89,227	15	0.68	0.38–1.23		29,137	4	0.62	0.21–1.77		0.67	0.40–1.11	
50–54	159,706	42		Referent		63,253			Referent			Referent	
≥55	35,001	7	0.67	0.30–1.49		20,114	7	1.53	0.61–3.85		0.96	0.52–1.75	
* p* for trend				0.84					0.59				
Surgical menopause													
Bilateral oophorectomy^d^	54,645	12	0.84	0.43–1.63		687	0	–			–		
Hysterectomy^d^	100,694	36	2.25	1.38–3.65		15,809	5	1.11	0.39–3.13		1.98	1.27–3.09	
Missing	131,173	30				7837	5						
Fertile Duration													
All women, per year increase	341,659	80	1.02	0.97–1.07		135,777	34	0.97	0.91–1.03		1.00	0.96–1.04	
Menopausal hormone therapy (MHT)													
Never	259,833	65		Referent		136,849	23		Referent			Referent	
Ever use	340,563	83	1.12	0.80–1.56		72,057	21	1.05	0.56–1.96		1.10	0.82–1.49	
Missing	5,795	1				556	0						
Timing of use													
Never	241,746	63		Referent		136,849	23		Referent			Referent	
Former	81,684	22	0.92	0.56–1.50		63,593	19	1.05	0.55–2.01		0.97	0.65–1.43	
Current	227,895	59	1.26	0.87–1.84		8464	2	1.04	0.24–4.47		1.25	0.87–1.78	
Missing	54,866	5				556	0						
Duration of use													
None	130,549	36		Referent		136,849	23		Referent			Referent	
<5 years	55,378	11	0.79	0.42–1.52		23,845	5	0.84	0.31–2.26		0.80	0.47–1.37	
5–9 years	37,442	12	1.30	0.66–2.55		16,452	2	0.45	0.10–1.97		1.09	0.59–2.02	
10+ years	66,548	16	0.72	0.35–1.47		13,717	7	1.53	0.62–3.76		0.97	0.55–1.70	
Missing	231,499	58				18,599	7						
* p* for trend				0.64					0.05				
MHT type^e^													
None	153,760	35		Referent		136,849	23		Referent			Referent	
Oestrogen only	125,090	38	1.44	0.90–2.31		2708	1	1.45	0.19	10.85	1.44	0.91–2.28	
Combination	100,318	22	1.01	0.58–1.76		5735	1	0.79	0.11–5.95		0.99	0.58–1.69	
Other MHT	15,982	1	0.34	0.05–2.54									
Missing	196,844	44				64,149	19						
MHT pill usage^e^													
None	87,966	24		Referent		136,849	23		Referent			Referent	
Used pills	118,376	36	1.06	0.63–1.79		6,501	2	1.34	0.31–5.76		1.09	0.67–1.78	
Other MHT	8,467	1	0.55	0.07–4.10		1879	0	–					
Missing	377,185	79				64,233	19						

^a^Numbers are for women with non-missing covariates.

^b^Adjusted for age (continuous), alcohol (g/day: none, ≤1.08, >1.08–3.58, >3.58–13.54, >13.54), BMI (kg/m^2^: <25, 25–29.9, ≥30), diabetes (yes, no), race (white, other), smoking (never, former, current), parent cohort study, menopausal status (pre-, post-menopausal), and education (<high school, high school, some college/vocational, college, graduate degree).

^c^Adjusted for age (continuous), alcohol (never, former, current light/occasional (<16 g/day), current heavy (≥16 g/day)), BMI (kg/m^2^: <25, 25–29.9, ≥30), smoking (never, former, current), menopausal status (pre-, post-menopausal), and education (<secondary school, secondary school, college, graduate degree).

^d^Reference group for oophorectomy and hysterectomy is females who had natural menopause aged between 50 and 54 years old.

^e^Information on MHT type and pill usage only available for current MHT users at baseline in the UK Biobank.

Among the 47 ICC cases and 98 controls evaluated for HBV and HCV infections, one case (2.1%) and no controls were positive for HBsAg. For anti-HCV, no cases and three controls (3.1%) were positive. The viral results were not incorporated into the main analyses, as the results were only available for a small proportion of LCPP participants. In sensitivity analyses that dropped HBsAg(+) and anti-HCV(+) cases, the results did not differ from the analyses that included all cases (data not shown). Similarly, analyses that removed cases that developed in the first two years of follow-up were similar to those presented (data not shown).

## Discussion

In the present study, long-term oral contraceptive use was associated with a 62% increased ICC risk, and hysterectomy was associated with a doubling of risk. The other reproductive factors were not associated with risk of ICC.

This is the first study to date to examine exogenous hormone use, reproductive factors and ICC risk. Prior studies have been limited to examination of all primary liver cancer or HCC only, which is the dominant form of liver cancer and accounts for 75% of primary liver cancer cases.^7^ Thus, all prior examinations of exogenous hormone use and reproductive risk factors for liver cancer have been primarily driven by the aetiology of HCC. However, we discuss these prior results herein to highlight the similarities and differences in the aetiology of these two types of liver cancer. HCC is 2–3 times more common among men than women,^34^ while incidence rates of ICC are only 30% higher in men than in women.^35^ Reasons for reduced sex differences in ICC risk are unclear, but may be partially explained by oestrogenic factors increasing risk in women, as reported in the current study.

In 1999, the International Agency for Research on Cancer (IARC) concluded that there was sufficient evidence that oral contraceptives increased risk of liver cancer in the absence of viral infections.^36^ However, a meta-analysis reported a 55% increased risk of liver cancer only in case-control studies, but no association in cohort studies.^37^ The most recent 2018 IARC monograph concluded that there was still sufficient evidence that oestrogen-progesterone combination oral contraceptives cause liver cancer.^38^ While the recent IARC monograph acknowledged that there was no association found in cohort studies, the majority of these to-date have included small numbers of cases. The prior study of HCC in the LCPP showed an increased, but non-significant, risk of HCC with more than 6 years of oral contraceptive use,^39^ which is similar to the significant increased risk of ICC reported herein for nine or more years of oral contraceptive use.

The reported associations between MHT use and primary liver cancer risk have been inconsistent. A recent meta-analysis reported that MHT was associated with a 40% decreased risk of primary liver cancer across five studies.^40^ Inverse associations were also reported for oestrogen-only, as well as oestrogen-progesterone combination MHT. However, there was significant heterogeneity between the studies in the meta-analysis. One of these studies examined MHT from prescription records and reported a 42% decreased risk of liver cancer.^41^ However, none of these studies were able to examine ICC independent of primary liver cancer. Our study reported that oestrogen-only MHT use in post-menopausal women was associated with a possible indication of increased risk of ICC, albeit non-significant, which was consistent in the cohorts in North America and the UK. However, the sample size was limited, and the UK Biobank only assessed type of MHT for women who reported ‘current’ MHT use at time of the questionnaire.

Experimental evidence suggests that oestrogen, potentially mediated through interleukin-6 (IL-6)^10^ or vascular endothelial growth factor (VEGF),^8^ promotes cholangiocarcinogenesis, while selective oestrogen receptor modulators can inhibit growth.^8–10^ Cholangiocytes can express both oestrogen receptor (ER)-α and -β, whereas hepatocytes express only ER-α.^42^ In bile duct ligated rats, ER-β increased 5-fold in cholangiocytes, whereas ER-α decreased in both cholangiocytes and hepatocytes.^42^ Thus, the increased risk of ICC associated with oestrogen-only MHT use is biologically plausible through modulation of cholangiocyte proliferation.^43,44^ While statistical power was still somewhat limited to examine this hypothesis, both the LCPP and the UK Biobank reported nearly identical effect estimates for the oestrogen-only MHT—ICC association.

In epidemiologic investigations of circulating sex steroid hormones and ICC risk, two studies have reported higher levels of circulating oestradiol in both men and women cholangiocarcinoma cases compared to controls.^11,12^ One of these studies was based in the LCPP and reported that a doubling of circulating oestrogen levels in women was associated with a 40% increased ICC risk.^12^ Neither study reported associations with circulating androgen levels.

Of the reproductive factors, which have been utilised as proxies of hormone status, parity is the most well studied in relation to primary liver cancer but there is significant heterogeneity in results. A recent meta-analysis reported a non-linear association, with a J-shaped relationship between parity and primary liver cancer.^40^ This restricted cubic spline model suggested that risk of primary liver cancer began to increase with more than five live births. Little to no association has been reported with age at menarche, age at first birth and age at menopause. However, two studies have reported oophorectomy is associated with an increased risk of HCC.^39,45^ Similarly, in two recent studies, medically recorded bilateral oophorectomy was associated with a 30–70% increased risk of non-alcoholic fatty liver disease.^46,47^ While the current study did not find an association with oophorectomy, there was an increased risk of ICC associated with hysterectomy. This could be due either to misclassified self-reported hysterectomy and oophorectomy status,^48^ whereby women that had an oophorectomy tend to misreport (i.e., report hysterectomy instead of oophorectomy), or to alterations in sex steroid hormones, particularly decreased androgen levels, in hysterectomised women.^49^ Additionally, women undergoing hysterectomy may be more likely to begin taking MHT.^50^ We also report that MHT use, in particular oestrogen-only MHT use, is more common among women who report hysterectomy than in those who report natural menopause. However, the LCPP does not have information on age at hysterectomy. In the UK Biobank, only 26% of participants started taking MHT after hysterectomy (7% within 1-year post-hysterectomy); thus, MHT initiation is not strongly related to hysterectomy. Further, adjustment for MHT use did not substantially change the hysterectomy-ICC association. Alternatively, hysterectomy, which has been associated with weight gain and diabetes, may have indirect effects on ICC risk.^51,52^ In a recent meta-analysis, we reported that excess adiposity and diabetes were both associated with a 50% increased ICC risk.^53^ Thus, hysterectomy could be leading to weight gain or development of diabetes in women that places them at higher ICC risk.

The current report is the first study focused specifically on reproductive factors and ICC. The large population of over 1.1 million women available from combining the LCPP and the UK Biobank allowed for investigation of reproductive factors and exogenous hormonal exposures in relation to ICC risk, which is a rare tumour with incidence rates typically 1.0/100,000 or less. Further, as the baseline enrolment for cohorts in the LCPP was 1980–1998 and in the UK Biobank was 2006–2010, the associations reported in both studies suggest that secular trends did not have influential effects. This study included a wide geographic representation from North America and the UK. Additionally, the prospective design minimises recall bias. In addition, sensitivity analyses that excluded ICCs that developed in the first two years of follow-up supported the results of the main analysis.

Limitations include exposure capture, risk factor information, and generalisability. All exposures in the included cohorts were self-reported. Thus, some of the exposures, for example oophorectomy, may not be reported accurately compared to medical report. However, the majority of studies to date have relied on self-reported data. In particular, for oral contraceptive use, there are not currently good resources with prescription information and sufficient follow-up for any liver cancer outcome. Formulations of oral contraceptives have changed over time, which makes examining and definitively addressing the association between oral contraceptive use and ICC challenging. Additionally, there was no information on specific MHT formulations, and for the UK Biobank, type of MHT used was only available for women currently reporting MHT use at the time of questionnaire administration. As serum or plasma samples were only available for a small number of ICC cases, we were unable to include HBV or HCV as potential covariates. However, the prevalence of HBV and HCV in the general population of women in the US and UK is exceedingly low (≤1%).^54–56^ Further, the UK Biobank is in the process of testing all participants for viral factors but the data are not yet publicly available. As these cohorts were established to examine all cancer types, and not specifically liver cancer, there is no information on pre-existing liver disease among the participants in the LCPP. Models are adjusted for diabetes, but diabetes type (1 or 2) is not captured in the majority of LCPP cohorts. However, type 2 diabetes accounts for 95% of diabetes diagnoses.^57^ As this is an older population, diabetes is utilised as a proxy of type 2 diabetes. Finally, this is a population of primarily white post-menopausal women. Thus, the generalisability of these results to other racial/ethnic groups may not be assumed.

In summary, we report that long-term oral contraceptive use and hysterectomy are associated with an increased risk of ICC. Other reproductive factors were unrelated to risk. While intriguing, replication of these findings is warranted, ideally in populations with medical record data to avoid potential misclassification of exposures.

## Supplementary information

Supplemental Tables S1 and S2

## Data Availability

Data can be obtained for legitimate research purposes. Access to LCPP data can be provided by submitting a request to Katherine McGlynn (mcglynnk@mail.nih.gov) or Peter Campbell (peter.campbell@cancer.org). UK Biobank data can be provided by submitting an application to the UK Biobank (https://www.ukbiobank.ac.uk/).

## References

[1] Saha SK, Zhu AX, Fuchs CS, Brooks GA (2016). Forty-year trends in cholangiocarcinoma Incidence in the U.S.: intrahepatic disease on the rise. Oncologist.

[2] Surveillance, Epidemiology, and End Results (SEER) Program (https://www.seer.cancer.gov) SEER*Stat Database: National Program of Cancer Registries (NPCR) and SEER Incidence - U.S. Cancer Statistics Public Use Database, Nov 2018 Sub (2001–2016), National Cancer Institute, DCCPS, Surveillance Research Program, released April 2019, based on the November 2018 submission.

[3] Altekruse SF, Devesa SS, Dickie LA, McGlynn KA, Kleiner DE (2011). Histological classification of liver and intrahepatic bile duct cancers in SEER registries. J. registry Manag..

[4] Alvaro D, Alpini G, Onori P, Franchitto A, Glaser SS, Le Sage G (2002). Alfa and beta estrogen receptors and the biliary tree. Mol. Cell Endocrinol..

[5] Alvaro, D., Alpini, G., Onori, P., Franchitto, A., Mancino, M. G., Glaser, S. et al. *Estrogen Regulation of Cholangiocyte Proliferation. Madame Curie Bioscience Database*. (Landes Bioscience, Austin, 2000).

[6] Alvaro D, Barbaro B, Franchitto A, Onori P, Glaser SS, Alpini G (2006). Estrogens and insulin-like growth factor 1 modulate neoplastic cell growth in human cholangiocarcinoma. Am. J. Pathol..

[7] London W. T., Petrick J. L., McGlynn K. A. Liver Cancer. in *Schottenfeld and Fraumeni cancer epidemiology and prevention*, 4th edn (eds Thun M. J., Linet M. S., Cerhan J. R. & Haiman C., Schottenfeld D.) pp xix, 1308 pages. (Oxford University Press: New York, 2018) pp xix, 1308 pages.

[8] Mancino A, Mancino MG, Glaser SS, Alpini G, Bolognese A, Izzo L (2009). Estrogens stimulate the proliferation of human cholangiocarcinoma by inducing the expression and secretion of vascular endothelial growth factor. Dig. Liver Dis..

[9] Sampson LK, Vickers SM, Ying W, Phillips JO (1997). Tamoxifen-mediated growth inhibition of human cholangiocarcinoma. Cancer Res..

[10] Isse K, Specht SM, Lunz JG, Kang LI, Mizuguchi Y, Demetris AJ (2010). Estrogen stimulates female biliary epithelial cell interleukin-6 expression in mice and humans. Hepatology.

[11] Hunsawong T, Singsuksawat E, In-chon N, Chawengrattanachot W, Thuwajit C, Sripa B (2012). Estrogen is increased in male cholangiocarcinoma patients’ serum and stimulates invasion in cholangiocarcinoma cell lines in vitro. J. Cancer Res Clin. Oncol..

[12] Petrick J. L., Florio A. A., Zhang X., Zeleniuch-Jacquotte A., Wactawski-Wende J., Van Den Eeden S. K. et al. Associations between Prediagnostic concentrations of circulating sex steroid hormones and liver cancer among post-menopausal women. *Hepatology*. 10.1002/hep.31057 (2019). 10.1002/hep.31057 PMC7391790 31808181

[13] McGlynn K. A., Sahasrabuddhe V. V., Campbell P. T., Graubard B. I., Chen J., Schwartz L. M. et al. Reproductive factors, exogenous hormone use and risk of liver cancer among U.S. women: results from the Liver Cancer Pooling Project. *Br J Cancer.* **112**, 1266–1272 (2015). 10.1038/bjc.2015.58 PMC4385955 25742475

[14] Simin J, Tamimi R, Lagergren J, Adami HO, Brusselaers N (2017). Menopausal hormone therapy and cancer risk: an overestimated risk?. Eur. J. Cancer.

[15] Schatzkin A, Subar AF, Thompson FE, Harlan LC, Tangrea J, Hollenbeck AR (2001). Design and serendipity in establishing a large cohort with wide dietary intake distributions: the National Institutes of Health-American Association of Retired Persons Diet and Health Study. Am. J. Epidemiol..

[16] Alavanja MC, Sandler DP, McMaster SB, Zahm SH, McDonnell CJ, Lynch CF (1996). The agricultural health study. Environ. Health Perspect..

[17] Flood A, Velie EM, Chaterjee N, Subar AF, Thompson FE, Lacey JV (2002). Fruit and vegetable intakes and the risk of colorectal cancer in the Breast Cancer Detection Demonstration Project follow-up cohort. Am. J. Clin. Nutr..

[18] Kramer BS, Gohagan J, Prorok PC, Smart C (1993). A National Cancer Institute sponsored screening trial for prostatic, lung, colorectal, and ovarian cancers. Cancer.

[19] Rexrode KM, Lee IM, Cook NR, Hennekens CH, Buring JE (2000). Baseline characteristics of participants in the Women’s Health Study. J. women’s health Gend.-based Med..

[20] Toniolo PG, Pasternack BS, Shore RE, Sonnenschein E, Koenig KL, Rosenberg C (1991). Endogenous hormones and breast cancer: a prospective cohort study. Breast Cancer Res. Treat..

[21] Calle EE, Rodriguez C, Jacobs EJ, Almon ML, Chao A, McCullough ML (2002). The American Cancer Society Cancer Prevention Study II Nutrition Cohort: rationale, study design, and baseline characteristics. Cancer.

[22] Munger RG, Folsom AR, Kushi LH, Kaye SA, Sellers TA (1992). Dietary assessment of older Iowa women with a food frequency questionnaire: nutrient intake, reproducibility, and comparison with 24-hour dietary recall interviews. Am. J. Epidemiol..

[23] Rosenberg L, Adams-Campbell L, Palmer JR (1995). The Black Women’s Health Study: a follow-up study for causes and preventions of illness. J. Am. Med. Women’s Assoc..

[24] Anderson GL, Manson J, Wallace R, Lund B, Hall D, Davis S (2003). Implementation of the Women’s Health Initiative study design. Ann. Epidemiol..

[25] Belanger CF, Hennekens CH, Rosner B, Speizer FE (1978). The nurses’ health study. Am. J. Nurs..

[26] Rohan TE, Soskolne CL, Carroll KK, Kreiger N (2007). The Canadian Study of Diet, Lifestyle, and Health: design and characteristics of a new cohort study of cancer risk. Cancer Detect Prev..

[27] Steering Committee of the PHS Research Group. (1989). Final report on the aspirin component of the ongoing Physicians’ Health Study. N. Engl. J. Med..

[28] Grobbee DE, Rimm EB, Giovannucci E, Colditz G, Stampfer M, Willett W (1990). Coffee, caffeine, and cardiovascular disease in men. N. Engl. J. Med..

[29] Boice JD, Mandel JS, Doody MM, Yoder RC, McGowan R (1992). A health survey of radiologic technologists. Cancer.

[30] Sudlow C, Gallacher J, Allen N, Beral V, Burton P, Danesh J (2015). UK biobank: an open access resource for identifying the causes of a wide range of complex diseases of middle and old age. PLoS Med..

[31] Catsburg C, Kirsh VA, Soskolne CL, Kreiger N, Bruce E, Ho T (2014). Associations between anthropometric characteristics, physical activity, and breast cancer risk in a Canadian cohort. Breast cancer Res. Treat..

[32] The SURVEYPHREG Procedure. in *SAS/STAT® 14.3 User’s Guide*. (SAS Institute Inc., Cary, 2017).

[33] Rothman, K. J., Greenland, S. & Lash, T. L. *Modern epidemiology. Wolters Kluwer Health/*. 3rd edn. (Lippincott Williams & Wilkins, Philadelphia, 2008).

[34] McGlynn KA, Petrick JL, London WT (2015). Global epidemiology of hepatocellular carcinoma: an emphasis on demographic and regional variability. Clin. Liver Dis..

[35] Van Dyke AL, Shiels MS, Jones GS, Pfeiffer RM, Petrick JL, Beebe-Dimmer JL (2019). Biliary tract cancer incidence and trends in the United States by demographic group, 1999-2013. Cancer.

[36] IARC. *Hormonal contraception and post-menopausal hormonal therapy 72* (IARCPress, Lyon, 1999).

[37] An N (2015). Oral contraceptives use and liver cancer risk: a dose-response meta-analysis of observational studies. Medicine.

[38] IARC. *Combined Estrogen-Progesterone Contraceptives 100A*. (IARCPress, Lyon, 2018).

[39] McGlynn KA, Sahasrabuddhe VV, Campbell PT, Graubard BI, Chen J, Schwartz LM (2015). Reproductive factors, exogenous hormone use and risk of hepatocellular carcinoma among US women: results from the Liver Cancer Pooling Project. Br. J. Cancer.

[40] Zhong GC, Liu Y, Chen N, Hao FB, Wang K, Cheng JH (2016). Reproductive factors, menopausal hormone therapies and primary liver cancer risk: a systematic review and dose-response meta-analysis of observational studies. Hum. Reprod. Update.

[41] McGlynn KA, Hagberg K, Chen J, Braunlin M, Graubard BI, Suneja N (2016). Menopausal hormone therapy use and risk of primary liver cancer in the clinical practice research datalink. Int J. Cancer.

[42] Alvaro D, Alpini G, Onori P, Perego L, Svegliata Baroni G, Franchitto A (2000). Estrogens stimulate proliferation of intrahepatic biliary epithelium in rats. Gastroenterology.

[43] Mancinelli R, Onori P, Demorrow S, Francis H, Glaser S, Franchitto A (2010). Role of sex hormones in the modulation of cholangiocyte function. World J. Gastrointest. Pathophysiol..

[44] Alvaro D, Mancino MG, Onori P, Franchitto A, Alpini G, Francis H (2006). Estrogens and the pathophysiology of the biliary tree. World J. Gastroenterol..

[45] Yu MW, Chang HC, Chang SC, Liaw YF, Lin SM, Liu CJ (2003). Role of reproductive factors in hepatocellular carcinoma: Impact on hepatitis B- and C-related risk. Hepatology.

[46] Matsuo K, Gualtieri MR, Cahoon SS, Jung CE, Paulson RJ, Shoupe D (2016). Surgical menopause and increased risk of nonalcoholic fatty liver disease in endometrial cancer. Menopause.

[47] Florio A. A., Graubard B. I., Yang B., Thistle J. E., Bradley M. C., McGlynn K. A. et al. Oophorectomy and risk of non-alcoholic fatty liver disease and primary liver cancer in the Clinical Practice Research Datalink. *Eur. J. Epidemiol.*10.1007/s10654-019-00526-1 (2019). 10.1007/s10654-019-00526-1 PMC6761000 31165323

[48] Phipps AI, Buist DS (2009). Validation of self-reported history of hysterectomy and oophorectomy among women in an integrated group practice setting. Menopause.

[49] Laughlin GA, Barrett-Connor E, Kritz-Silverstein D, von Muhlen D (2000). Hysterectomy, oophorectomy, and endogenous sex hormone levels in older women: the Rancho Bernardo Study. J. Clin. Endocrinol. Metab..

[50] Haney AF, Wild RA (2007). Options for hormone therapy in women who have had a hysterectomy. Menopause.

[51] Moorman PG, Schildkraut JM, Iversen ES, Myers ER, Gradison M, Warren-White N (2009). A prospective study of weight gain after premenopausal hysterectomy. J. Women’s Health (Larchmt.).

[52] Appiah D, Winters SJ, Hornung CA (2014). Bilateral oophorectomy and the risk of incident diabetes in postmenopausal women. Diabetes Care.

[53] Petrick JL, Thistle JE, Zeleniuch-Jacquotte A, Zhang X, Wactawski-Wende J, Van Dyke AL (2018). Body mass index, diabetes and intrahepatic cholangiocarcinoma risk: The Liver Cancer Pooling Project and Meta-analysis. Am. J. Gastroenterol..

[54] Balogun MA, Ramsay ME, Hesketh LM, Andrews N, Osborne KP, Gay NJ (2002). The prevalence of hepatitis C in England and Wales. J. Infect..

[55] Roberts H, Kruszon-Moran D, Ly KN, Hughes E, Iqbal K, Jiles RB (2016). Prevalence of chronic hepatitis B virus (HBV) infection in U.S. households: National Health and Nutrition Examination Survey (NHANES), 1988-2012. Hepatology.

[56] Alter M. J., Kruszon-Moran D., Nainan O. V., McQuillan G. M., Gao F. X., Moyer L. A. et al. The prevalence of hepatitis C virus infection in the United States, 1988 through 1994. *N. Engl. J. Med.* **341**, 556–562 (1999). 10.1056/NEJM199908193410802 10451460

[57] Centers for Disease Control and Prevention. *National Diabetes Statistics Report, 2017*. (Centers for Disease Control and Prevention, U.S. Dept of Health and Human Services, Atlanta, 2017).

