# Supplementary material for: Exogenous hormone use, reproductive factors and risk of intrahepatic cholangiocarcinoma among women: results from cohort studies in the Liver Cancer Pooling Project and the UK Biobank
Source: Br J Cancer. 2020 May 7;123(2):316–24. doi: 10.1038/s41416-020-0835-5 (PMC7374167; doi:10.1038/s41416-020-0835-5)
Supplement: Supplementary file 1 — Supplemental Tables S1 and S2 [file 41416_2020_835_MOESM1_ESM.docx]

**Supplemental Table S2.** Distribution of menopausal hormone therapy (MHT) use among women who underwent natural menopause or hysterectomy.

|  | **LCPP** | |  | **UK Biobank** | |
| --- | --- | --- | --- | --- | --- |
| **Menopausal Hormone Therapy** | Natural  Menopause N (%) | Hysterectomy  N (%) |  | Natural  Menopause N (%) | Hysterectomy  N (%) |
| No MHT | 213,054 (57.8) | 31,779 (27.1) |  | 118,404 (95.6) | 14,499 (90.1) |
| Any MHT | 155,686 (42.2) | 85,411 (72.9) |  | 5,403 (4.4) | 1,602 (9.9) |
| **MHT Type** |  |  |  |  |  |
| No MHT | 120,699 (56.3) | 14,956 (24.0) |  | 70,838 (92.9) | 4,489 (73.7) |
| Estrogen Only | 28,701 (13.4) | 39,339 (63.1) |  | 1,041 (1.4) | 1,459 (24.0) |
| Combined Therapy | 64,886 (30.3) | 8,062 (12.9) |  | 4,345 (5.7) | 143 (2.3) |
